# Efficacy of single-dose HPV vaccination among young African women

**DOI:** 10.1056/EVIDoa2100056

**Published:** 2022-04-11

**Authors:** Ruanne V. Barnabas, Elizabeth R. Brown, Maricianah A. Onono, Elizabeth A. Bukusi, Betty Njoroge, Rachel L. Winer, Denise A. Galloway, Leeya F. Pinder, Deborah Donnell, Imelda Wakhungu, Ouma Congo, Charlene Biwott, Syovata Kimanthi, Linda Oluoch, Kate B. Heller, Hannah Leingang, Susan Morrison, Elena Rechkina, Stephen Cherne, Torin T. Schaafsma, R. Scott McClelland, Connie Celum, Jared M. Baeten, Nelly Mugo

**Affiliations:** 1 Department of Global Health, University of Washington, Seattle, USA; 2 Division of Allergy and Infectious Diseases, University of Washington, Seattle, USA; 3 Department of Epidemiology, University of Washington, Seattle, USA; 4 Department of Biostatistics, University of Washington, Seattle, USA; 5 Department of Obstetrics and Gynecology, University of Washington, Seattle, USA; 7 Department of Pathology, University of Washington, Seattle, USA; 8 Vaccine and Infectious Disease Division, Fred Hutchinson Cancer Research Center, Seattle, USA; 9 Public Health Sciences, Fred Hutchinson Cancer Research Center, Seattle, USA; 10 Human Biology Division, Fred Hutchinson Cancer Research Center, Seattle, USA; 11 Center for Microbiology Research, Kenya Medical Research Institute, Kenya; 12 Center for Clinical Research, Kenya Medical Research Institute, Kenya; 13 Gilead Sciences, Foster City, CA, USA; 14 Division of Infectious Diseases, Massachusetts General Hospital, Boston, MA, USA

## Abstract

**Background:**

Single-dose HPV vaccination, if efficacious, would be tremendously advantageous; simplifying implementation and decreasing costs.

**Methods:**

We performed a randomized, multi-center, double-blind, controlled trial of single-dose nonavalent (HPV 16/18/31/33/45/52/58/6/11) or bivalent (HPV 16/18) HPV vaccination compared to meningococcal vaccination among Kenyan women aged 15-20 years. Enrollment and six monthly cervical swabs and a month three vaginal swab were tested for HPV DNA. Enrollment sera were tested for HPV antibodies. The modified intent-to-treat (mITT) cohort comprised participants who tested HPV antibody negative at enrollment and HPV DNA negative at enrollment and month three. The primary outcome was incident persistent vaccine-type HPV infection by month 18.

**Results:**

Between December 2018 and June 2021, 2,275 women were randomly assigned and followed; 758 received the nonavalent HPV vaccine, 760 the bivalent HPV vaccine, and 757 the meningococcal vaccine; retention was 98%. Thirty-eight incident persistent infections were detected in the HPV 16/18 mITT cohort: one each among participants assigned to the bivalent and nonavalent groups and 36 among those assigned to the meningococcal group; nonavalent Vaccine Efficacy (VE) was 97.5% (95%CI 81.7-99.7%, p=<0.0001), and bivalent VE was 97.5% (95%CI 81.6-99.7%, p=<0.0001). Thirty-three incident persistent infections were detected in the HPV 16/18/31/33/45/52/58 mITT cohort: four in the nonavalent group and 29 in the meningococcal group; nonavalent VE for HPV 16/18/31/33/45/52/58 was 88.9% (95%CI 68.5-96.1%, p<0.0001). The rate of SAEs was 4.5-5.2% by group.

**Conclusions:**

Over the 18 month time-frame we studied, single-dose bivalent and nonavalent HPV vaccines were each highly effective in preventing incident persistent oncogenic HPV infection, similar to multidose regimens.

## Introduction

Almost 90% of the more than 600,000 new cervical cancer cases and 340,000 cervical cancer deaths in 2020 occurred in low- and middle-income countries (LMICs).^1^ Vaccination to prevent human papillomavirus (HPV) infection, the primary cause of cervical cancer, is a key intervention in the World Health Organization’s (WHO) Global Cervical Cancer Elimination Strategy, which calls for vaccination of 90% of girls.^2,3^ HPV vaccines, licensed as 2-3 intramuscular injections over the course of 6-12 months, reduce an individual’s risk of acquiring persistent oncogenic HPV infection by >90%.^4,5,6^ At the population level, increasing vaccine coverage increases effectiveness; vaccination of multi-age adolescent cohorts (9-14 years) with catch-up vaccination (to age 26 years) doubles the prevention of HPV-associated precancerous lesions.^7^ However, HPV vaccine coverage remains low;^8^ in 2019, the global coverage for HPV vaccination was 15% among adolescent girls^9^.

In LMICs, low vaccine coverage is due, in part, to the cost and logistics of reaching girls with standard multi-dose vaccine schedule; single-dose vaccination could halve vaccination costs, potentially increase coverage, and simplify the logistics compared to multidose administration. Currently, four HPV vaccines are licensed, all targeting high-risk (oncogenic) HPV types that cause 70% of cancers (HPV 16/18) and two also targeting low-risk HPV types that cause genital warts (HPV 6/11); the bivalent vaccine (Cervarix® and Cecolin®) prevents HPV 16/18 infection, the quadrivalent vaccine (Gardasil®) prevents HPV 16/18/6/11, and the nonavalent vaccine (Gardasil-9®) prevents HPV 16/18/31/33/45/52/58/6/11 infection (including five additional high-risk HPV types).^10^

Observational studies suggest that single-dose HPV vaccine effectiveness is equivalent to a two- or three-dose regimen: however, vaccination guidelines recommend multidose strategies and questions persist regarding single-dose efficacy.^11-14^ In Kenya, multidose HPV vaccination is offered to 9-14-year-old girls through the national immunization program from October 2019; however, due to supply constraints, HPV vaccination was offered to 10-year-old girls only. To date, an estimated 10% of 10 year old girls have received their first HPV vaccine dose and 3% have received the second dose.^15^ Catch-up vaccination for adolescent girls and young women 15 years of age and older is not provided, with cervical cancer screening offered to older women. Testing the efficacy of single-dose HPV vaccination among young women age 15 years and older, within the context of cytological screening for dysplastic lesions in a clinical trial, was determined to be ethical as vaccination for this age group in Kenya and many LMICs is not currently supported through national programs or global immunization bodies. Specifically, we evaluated zero versus single-dose HPV vaccination against the backdrop of substantial disparities in cervical cancer incidence.^16^ Also, a superiority design was chosen, compared to a non-inferiority design, as the smaller sample size and shorter time line would support robust, feasible, and timely evidence. Here we report the findings of an efficacy trial of single-dose bivalent and nonavalent HPV vaccination among young women in Kenya.

## Methods

### Trial design and Oversight

This randomized, multi-center, double-blind, parallel, three-arm controlled, superiority trial tested the efficacy of single-dose bivalent (HPV 16/18) and single-dose nonavalent (HPV 16/18/31/33/45/52/58/6/11) HPV vaccination, as described in the published protocol paper.^17^ Kenya’s HPV National Immunization Program, launched in October 2019, offers two doses of the quadrivalent HPV vaccine to 9-10-year-old girls and is not provided through the National Immunization Program for persons aged 15 years and older; meningococcal vaccination is used during outbreaks.^18^ Meningococcal vaccination was chosen as the comparator because meningococcal antibodies offer potential clinical benefits and do not impact HPV outcomes. Participants were randomized to 1) immediate nonavalent HPV vaccination and delayed (36 months after enrollment) meningococcal vaccination, 2) immediate bivalent HPV vaccination and delayed meningococcal vaccination, or 3) immediate meningococcal vaccination and delayed HPV vaccination. The primary analysis was planned for month 18, with the final analysis at month 36 evaluating durability (not reported herein). At this time the trial is ongoing. After the 18 month results presented herein, we are continuing follow-up in a blinded cross-over design to evaluate vaccine durability.

The Kenya Medical Research Institute (KEMRI) Scientific and Ethics Review Unit (SERU) and the University of Washington (UW) Institutional Review Board (IRB) approved the study. The study was registered with ClinicalTrials.gov (NCT03675256).

### Participants

Participants were recruited through community outreach. Participants were eligible for randomization if they were able to provide informed consent, age 15 to 20 years old, female sex assigned at birth, sexually active reporting one to five lifetime partners, and resident within the study area. Study exclusion criteria were a positive HIV diagnostic test, history of HPV vaccination, allergies to vaccine components or latex, current pregnancy, hysterectomy, or history of immunosuppressive conditions.

### Setting

The study was conducted at three KEMRI clinical sites in Thika, Nairobi, and Kisumu. All participants, and their parents/guardians in the case of minors, provided informed consent, which included counseling about randomization, risks and benefits of participation, study procedures, and their rights as research participants.

### Screening and enrollment

Potential participants completed eligibility screening with a provider including a detailed medical history, collection of external genital (labial/vulvar/perineal) and cervical swabs for HPV deoxyribonucleic acid (DNA) testing, and serum for HPV antibody testing. Participants received cytological cervical cancer screening at enrollment. Sexual and reproductive health services (contraception, sexually transmitted infection diagnosis and treatment, and HIV pre-exposure prophylaxis) were offered at enrollment and every visit. All questionnaires were conducted using electronic case report forms (DF/Net Research, Inc. ©, Seattle, WA, US).

### Randomization and Vaccination

Randomization was stratified by site, using a fixed block size of 15, and a 1:1:1 allocation. Study staff, participants, investigators, clinic staff, lab technicians, and other study team members did not have access to the randomization codes, except for the unblinded statistical analysts and unblinded pharmacists at each site. Blinded study assignment was implemented via http://www.randomize.net (Ottawa, ON, Canada). An unblinded pharmacist entered the participant identification number on randomize.net, obtained the next sequential intervention assignment, recorded the participant identification number and randomization identifier on an electronic case report form, drew up the vaccine in a masked syringe, and administered the vaccination.

### Study follow-up procedures

Participants were seen at months three, six, and then every six months for 18 months after enrollment. Providers administered clinical questionnaires and collected a cervical swab at each six-month visit. Participants self-collected vaginal swabs using validated instructions at month three; self-collected swabs, which have similar accuracy compared to provider collected cervical swabs,^19^ were available at subsequent follow-up visits by participant choice or to comply with COVID-19 research restrictions.

### Laboratory methods

HPV DNA genotyping was conducted using the Anyplex II HPV28 assay (Seegene, Seoul, South Korea), a multiplexed type-specific real-time polymerase chain reaction (PCR) based assay^20,21^ at the UW East Africa STI Laboratory, Mombasa, Kenya with standard proficiency testing.^22^ For HPV-positive samples, a low (+), intermediate (++), or high (+++) positivity was indicated; + or greater were considered positive. All runs included negative and positive controls, and the housekeeping human gene, β-globin, as an internal control. Runs were performed with CFX96 Real-time PCR System (BioRad, Hercules, California).

Serum specimens were shipped to the University of Washington, Seattle, WA, US, and tested at the Galloway Lab, Fred Hutchinson Cancer Research Center. HPV IgG antibodies were detected using a multiplex Luminex assay.^23,24^ The mean pre-established fluorescent intensity (MFI) seropositivity cutoffs for HPV 16/18/31/33/45/52/58 were used (Table S14).

Sexually transmitted infections (*Neisseria gonorrhoeae, Chlamydia trachomatis,* or *Trichomonas vaginalis*) were assessed by nucleic acid amplification testing (APTIMA; Hologic/GenProbe, San Diego, CA) at the University of Washington-University of Nairobi East Africa STI Laboratory; HSV-2 was evaluated by the Focus ELISA and bacterial vaginosis was evaluated using the Nugent Score.

### Outcomes and assessment

The primary trial endpoint was incident persistent cervical HPV infection among participants who tested HPV DNA negative (external genital and cervical swabs) at enrollment and month three (self-collected vaginal swab) and HPV antibody negative at enrollment (the modified intent-to-treat (mITT) cohort). For inclusion in the HPV 16/18 mITT cohort, participants were HPV 16/18 naïve. Similarly, for the HPV 16/18/31/33/45/52/58 mITT cohort, participants were HPV 16/18/31/33/45/52/58 naïve. Persistent HPV, a surrogate marker for cervical dysplasia/precancer, was defined as high-risk vaccine type specific HPV (i.e., HPV 16/18 for the bivalent vaccine and HPV 16/18/31/33/45/52/58 for the nonavalent vaccine) detected at two consecutive time points no less than four months apart after month three and up to and including month 18 (same HPV type at both time points) for the primary analysis.^5^ Participants without swabs post-month 3 did not contribute follow-up time in the primary analysis. Participants in the bivalent vaccine group were not included in the HPV 16/18/31/33/45/52/58 analysis as the study was not powered to detected cross-protection. Cervical swabs were tested for the primary endpoint; vaginal swabs were substituted if necessary. Sensitivity analysis was planned on the following subset: participants who tested HPV DNA negative at enrollment, month three, and month six and antibody negative at enrollment (extended sensitivity cohort) to match the analysis cohort for HPV vaccine licensure trials. The extended sensitivity cohort analysis used all available data, including visits after the pre-specified month 18 data cut. Safety was assessed through adverse event reporting following the United States National Institute of Allergy and Infectious Diseases guidelines.^25^

### Statistical analysis

Sample size calculations assumed that 52% of participants would meet requirements for inclusion in the mITT cohort based on the observed prevalence of HPV infection in similar settings.^26^ The sample size calculations also assumed a combined persistent HPV 16/18/31/33/45/52/58 annual incidence of 5%, single-dose vaccine efficacy of 75%, and loss-to-follow-up of 10% with a fixed follow-up time of 12 months. Assuming a proportional hazards model (seqDesign in R) with 80% power to detect 75% efficacy, a sample size of 2250 participants was planned.

We used Cox proportional hazards (PH) models stratified by site to estimate the hazard ratios (HRs) of the interventions versus control for the primary and sensitivity analyses. Models for the sensitivity analyses used crude incidence rate ratios instead of Cox when no events were observed in a group. Follow-up was calculated as days since the month three visit for the primary analysis, and days since month six for the extended sensitivity analysis. Participants who did not reach the efficacy endpoints were censored at the time of the last negative test at or before the month 18 visit. Vaccine efficacy was expressed as a 1 minus the hazard ratio (or relative risk). The log-rank test stratified by site was used to calculate the p-value. Cumulative incidence Kaplan-Meier curves of time to infection were calculated by intervention group. Efficacy analyses were performed on the month 18 mITT cohorts. In post-hoc analysis, we evaluated the absolute difference in cumulative incidence of HPV from the Kaplan-Meier curves at month 18. We calculated the cumulative incidence of chlamydia and gonorrhea during follow-up by assigned group.

Safety was assessed among all participants; the three groups were compared using Fisher’s exact test. We performed all analyses using SAS software, version 9.2 (SAS Institute, North Carolina, US) and double coded in R (version 4.1).

An independent Data Safety and Monitoring Board (DSMB) was constituted to review study progress, participant safety, and the primary outcome; the DSMB met annually.

## Results

### Participants

**Figure 1:**
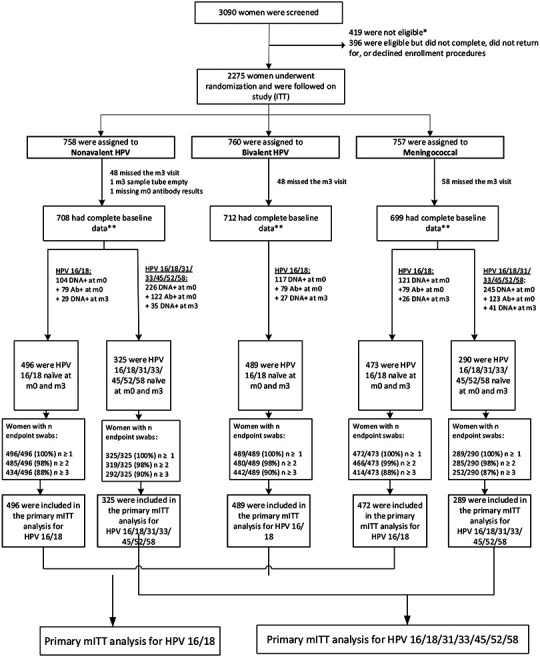
Randomized trial profile *of the 419 people who were ineligible for randomization, 132 (16%) had a positive pregnancy test, 51 (6%) were not willing to follow study procedures or be randomized, 34 (4%) had a positive rapid HIV diagnostic test, and 172 (21%) met other exclusion criteria. **Complete baseline data includes HPV antibody results at month 0 and HPV DNA results at month 0 and month 3.

Between December 20^th^, 2018, and November 15^th^, 2019, 3,090 participants were screened for study eligibility and 2,275 (74%) were enrolled. Of those ineligible, 132 (16%) had a positive pregnancy test, 51 (6%) declined study procedures, 34 (4%) had a positive rapid HIV test, and 172 (21%) met other exclusion criteria. Enrolled participants were randomized (Figure 1): 758 to the nonavalent HPV vaccine group, 760 to the bivalent HPV vaccine group, and 757 to the meningococcal vaccine group. At enrollment, 57% of participants (n=1,301) were age 15 to 17 years, and 61% (n=1,392) had one lifetime sexual partner with comparable baseline characteristics between the groups (Table S1). The group was representative of the population who would be eligible for HPV vaccination in this manner should such a decision be made - see Table S22 in the supplemental appendix.

For HPV 16/18, participants who tested HPV 16/18 antibody or HPV 16/18 DNA positive at enrollment or HPV DNA positive month three (n=661), or had missing antibody results (n=1) or a missing month three swab (n=155) were excluded. Among the 1,458 participants meeting criteria for the primary HPV 16/18 mITT analysis, 496 were in the nonavalent, 489 in the bivalent, and 473 in the meningococcal group. For HPV 16/18/31/33/45/52/58, participants who tested HPV 16/18/31/33/45/52/58 antibody or HPV 16/18/31/33/45/52/58 DNA positive at enrollment or HPV DNA positive at month three (n=792) or had missing antibody results (n=1) or a missing month three swab (n=106) were excluded. Of the 615 participants eligible for the primary HPV 16/18/31/33/45/52/58 analysis, 325 were in the nonavalent and 290 in the meningococcal vaccine group. One participant in the meningococcal vaccine group did not have at least one post-month three endpoint swab. The median age was 17 years for the HPV 16/18 and HPV 16/18/31/33/45/52/58 mITT cohorts ; and, overall, the baseline characteristics by study groups were comparable (Tables 1 and S27).

One hundred percent of participants received their assigned vaccine, without administration error. By the month 18 visit, retention for assessment of the primary endpoints was 98% for two swabs and 94% for three swabs; 94% of swabs were cervical swabs and 6% of swabs were self-collected vaginal swabs (Tables S5-8, S13). The cumulative incidence of chlamydia, gonorrhea, and persistent non-vaccine HPV types was comparable across the three study groups (Tables S16 and S26).

### Primary outcome

A total of 38 incident persistent infections were detected in the HPV 16/18 mITT cohort: one each among participants assigned to the bivalent and nonavalent vaccine groups and 36 among those assigned to the meningococcal vaccine group (Table 2a). The incidence of persistent HPV 16/18 was 0.17/100 woman-years in the bivalent and nonavalent vaccine groups, compared to 6.83/100 woman-years in the meningococcal vaccine control group. Bivalent Vaccine Efficacy (VE) was 97.5% (95% CI 81.7-99.7%, p=<0.0001) and nonavalent VE was 97.5% (95% CI 81.6-99.7%, p=<0.0001) (Figure 2a). Thirty-three incident persistent infections were detected in the HPV 16/18/31/33/45/52/58 mITT cohort: four in the nonavalent vaccine group and 29 in the meningococcal vaccine group (Table 2b). The incidence of persistent HPV 16/18/31/33/45/52/58 was 1.03/100 woman-years in the nonavalent vaccine group compared to 9.42/100 woman-years in the meningococcal group. Nonavalent VE for HPV 16/18/31/33/45/52/58 was 88.9% (95% CI 68.5-96.1%, p<0.0001) (Figure 2b).

In the extended sensitivity analysis, there were a total of 16 incident persistent infections in the HPV 16/18 mITT cohort: 0 each among participants assigned to the bivalent and nonavalent vaccine groups and 16 among those assigned to the meningococcal vaccine group (Table S9). HPV 16/18 incidence was 0 per 100 women-years in the nonavalent and bivalent vaccine groups and 3.9 per 100 women years in the meningococcal control group; nonavalent VE was 100% (p<0.0001) and bivalent VE was 100% (p<0.0001) (Table S9). In the extended sensitivity analysis, there were a total of 15 incident persistent infections in the HPV 16/18/31/33/45/52/58 mITT cohort: one among participants assigned to the nonavalent group and 14 among those assigned to the meningococcal group; nonavalent VE was 95.0% (95% CI 62.1-99.4%, p=<0.0001) (Table S10). Vaccine efficacy results were similar in the sensitivity analysis including participants with HPV antibodies at enrollment (Tables S23 and S24)

In *post-hoc* analysis, using only provider collected endpoint cervical swabs and excluding self-collected vaginal swabs, the results for the primary analysis were not different: the VE was 97.3% (95% CI 80.0-99.6 %) for each of the bivalent and nonavalent vaccines in the HPV 16/18 mITT cohort. Nonavalent vaccine efficacy was 91.4% (95% CI 71.8-97.4%) in the HPV 16/18/31/33/45/52/58 mITT cohort (Tables S11-12).

In *post-hoc* analysis, the absolute reduction in the HPV 16/18 mITT cohort for cumulative incident persistent HPV 16/18 infection was −7.7% (95% CI −10.4 – −5.0%) for both the bivalent and nonavalent vaccines; an absolute incidence of 0.2% (95% CI 0.0 – 0.6%) in the bivalent and nonavalent vaccine groups compared to 7.9% (95% CI 5.4 – 10.4%) in the meningococcal group. For the HPV 16/18/31/33/45/52/58 mITT cohort, the absolute reduction in persistent HPV 16/18/31/33/45/52/58 infection was −9.3% (95% CI −13.6 – −5.1%) for the nonavalent vaccine; an absolute incidence of 1.3% (95% CI 0.0 – 2.5%) in the nonavalent vaccine group compared to 10.6% (95% CI 6.9 – 14.2%) in the meningococcal group.

### Safety

There were 112 participants who experienced serious adverse events (SAEs), which included 57 participants with pregnancy-related SAEs, 46 with infections or inflammatory conditions (of which 31 were malaria), seven injuries, and five mental health illnesses. Overall, the frequency was similar between groups (Table 3). There was one death in the study as a result of a septic abortion and systemic sepsis. SAEs were assessed as not related to the study vaccines. Five participants had abnormal cytology at enrollment, which were all followed until the lesions resolved or the participant received treatment. Social harms were reported by 0.09% of participants (n=2) and included lack of social support from friends and family for trial participation.

## Discussion

Over the 18 months of this trial, the efficacy of single-dose bivalent or nonavalent HPV vaccine was very high among Kenyan adolescent girls and young women, demonstrating high levels of protection against vaccine-specific oncogenic HPV infection. Protection against HPV 16/18 infection was 97.5% for both vaccines; together with observed high reductions in the absolute cumulative incidence this suggests, should the protection have a durable effect, the potential for public health impact in the context of disparities in outcomes for cervical cancer cases and deaths (Table S22). Saliently, we were able to exclude single-dose HPV 16/18 vaccine efficacy less than 81%, the lower limit of the confident interval for both vaccines. Overall, the rate of HPV infection in this population of African adolescent girls and young women was high – 9.42 per 100 woman-years in the control group, approximately a third higher than in previous trials, highlighting the need for effective, scalable vaccine programs that can achieve high coverage and reduce this high incidence of HPV infection and potential cervical cancer.^4,27^ The high level of efficacy builds on observational data^11,12^ and provides, should the effect be sustained, evidence for single-dose HPV vaccination to prevent persistent HPV infections, which could increase vaccine access and coverage, offering a cost-effective strategy for cervical cancer prevention.^28^

Strengths of the study include the randomized, double-blind, controlled design, high retention, measurement of cervical HPV DNA as the outcome, determination of persistent HPV DNA, and the head-to-head comparison of the licensed bivalent and nonavalent HPV vaccines in protection against persistent infection with oncogenic HPV types included in the vaccines. In addition, the trial successfully enrolled persons exposed to HPV infection who were successfully retained in all randomized groups, allowing rapid assessment of single-dose efficacy.

We acknowledge that the study has limitations. First, the duration of follow-up is 18 months and the durability of single-dose vaccine efficacy remains to be demonstrated. However, observational data for single-dose HPV vaccination supports efficacy over a decade.^11^ Following these results, participants will receive blinded cross-over^29^ vaccination, ensuring all receive HPV vaccination, with an additional 18 months follow-up to evaluate single-dose durability, and access to the second dose following guidelines. The blinded cross-over design will allow us to calculate the durability of the vaccine efficacy demonstrated to date. Second, the proportion of randomized participants who were naive to HPV 16/18/31/33/45/52/58 was lower than expected (~40%) potentially decreasing the study power; however, incidence was higher than assumed and the efficacy result is statistically significant. Third, 6% of primary endpoint swabs were self-collected, and 94% were provider collected. Ideally, collection would be identical; however, the correlation between self-collected vaginal and provider collected cervical swabs is high^19^ and there was no difference in the results when self-collected swabs were excluded. An additional concern is whether antibody levels were declining over the observation period such that the high efficacy initially observed would be sustained. However, in a study conducted in India over ten years duration, antibody levels at plateau were such that vaccine efficacy is high (>95%)^11^ suggesting that even higher antibody levels could only demonstrate a small further increase in vaccine efficacy. In addition, the plateau level for single-dose HPV vaccination is reached by month 12.^30^ Lastly, while the GST-ELISA multiplex assay used to exclude participants with HPV antibodies at enrollment demonstrated overall agreement of 89% with the gold standard secreted alkaline phosphatase pseudovirion based neutralization assay,^31^ misclassification of participants as antibody naïve would not be different by study group. Further in sensitivity analysis including participants with HPV antibodies at baseline, overall vaccine efficacy was in keeping with the primary findings (Tables S23 and S24).

Cervical cancer is the fourth most common cancer among women globally, the second most frequent in sub-Saharan Africa and primarily affects women between ages 30-49 years and is the leading cause of cancer deaths in sub-Saharan Africa.^32,33^ Cervical cancer is almost entirely preventable through HPV vaccination. If the effects of single-dose HPV vaccination are durable, as we have reason to believe they will be, this approach could serve to close the gap between the WHO’s goal of 90% HPV vaccination coverage by 2030 and the 15% of girls globally currently vaccinated,^9,34^ alleviate vaccine supply constraints,^35^ and provide global policy makers with options to allocate existing HPV vaccine supply.

## Supplementary Material

Supplement

## Figures and Tables

**Figure 2a:**
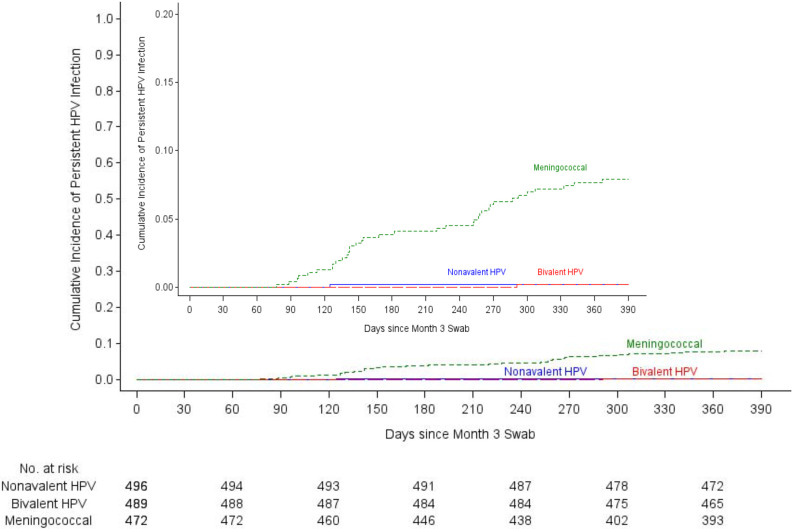
Kaplan-Meier curves for the primary, HPV 16/18 modified intention-to-treat analysis

**Figure 2b:**
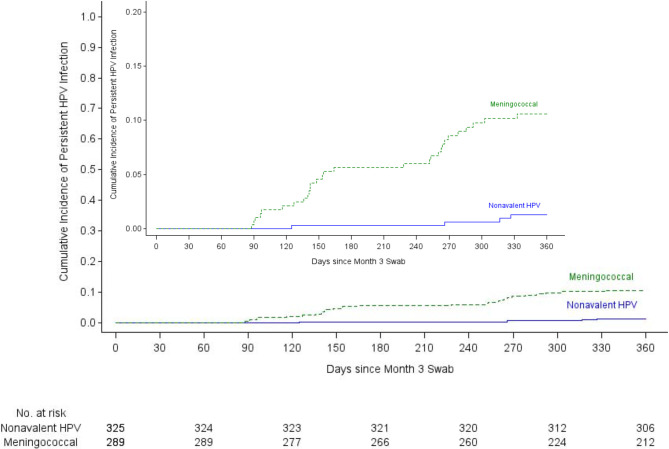
Kaplan-Meier curves for the primary, HPV 16/18/31/33/45/52/58 modified intention-to-treat analysis

**Table 1:** Baseline characteristics: modified intention-to-treat (mITT) Cohort

		HPV 16/18 mITT			HPV 16/18/31/33/45/52/58 mITT	
		Nonavalent HPV	Bivalent HPV	Meningococcal	Nonavalent HPV	Meningococcal
Characteristic	Category					
	Total	496	489	473	325	290
Age group (years)	15-17	299 (60.3%)	278 (56.9%)	278 (58.8%)	197 (60.6%)	168 (57.9%)
	18-20	197 (39.7%)	211 (43.1%)	195 (41.2%)	128 (39.4%)	122 (42.1%)
Marital status	Never married	478 (96.4%)	462 (94.5%)	446 (94.3%)	315 (96.9%)	269 (92.8%)
	Married	14 (2.8%)	24 (4.9%)	20 (4.2%)	7 (2.2%)	15 (5.2%)
	Previously Married	3 (0.6%)	3 (0.6%)	7 (1.5%)	2 (0.6%)	6 (2.1%)
	Other	1 (0.2%)	0 (0.0%)	0 (0.0%)	1 (0.3%)	0 (0.0%)
Education (highest level)	No schooling	1 (0.2%)	2 (0.4%)	1 (0.2%)	1 (0.3%)	1 (0.3%)
	Primary school, some or complete	40 (8.1%)	30 (6.1%)	36 (7.6%)	27 (8.3%)	27 (9.3%)
	Secondary school, some or complete	359 (72.4%)	368 (75.3%)	355 (75.1%)	241 (74.2%)	220 (75.9%)
	Post-secondary school	96 (19.4%)	89 (18.2%)	81 (17.1%)	56 (17.2%)	42 (14.5%)
Earns an income of her own	No	437 (88.1%)	417 (85.3%)	417 (88.2%)	284 (87.4%)	248 (85.5%)
	Yes	59 (11.9%)	72 (14.7%)	56 (11.8%)	41 (12.6%)	42 (14.5%)
Has a current main or steady sexual partner	No	144 (29.0%)	152 (31.1%)	145 (30.7%)	98 (30.2%)	95 (32.8%)
	Yes	352 (71.0%)	337 (68.9%)	328 (69.3%)	227 (69.8%)	195 (67.2%)
Age when first had vaginal intercourse (years)	<15	123 (24.8%)	116 (23.7%)	103 (21.8%)	80 (24.6%)	65 (22.4%)
	15-17	265 (53.4%)	274 (56.0%)	282 (59.6%)	185 (56.9%)	173 (59.7%)
	>18	96 (19.4%)	93 (19.0%)	79 (16.7%)	54 (16.6%)	46 (15.9%)
	Don't remember	12 (2.4%)	6 (1.2%)	9 (1.9%)	6 (1.8%)	6 (2.1%)
Lifetime number of sex partners	1	322 (64.9%)	332 (67.9%)	289 (61.1%)	217 (66.8%)	184 (63.4%)
	2	121 (24.4%)	100 (20.4%)	113 (23.9%)	78 (24.0%)	65 (22.4%)
	>3	53 (10.7%)	57 (11.7%)	71 (15.0%)	30 (9.2%)	41 (14.1%)
Condom use with last vaginal sex	No	153 (30.8%)	155 (31.7%)	140 (29.6%)	98 (30.2%)	78 (26.9%)
	Yes	237 (47.8%)	235 (48.1%)	238 (50.3%)	156 (48.0%)	144 (49.7%)
	No sex in past year	106 (21.4%)	99 (20.2%)	95 (20.1%)	71 (21.8%)	68 (23.4%)

The baseline characteristics of the intention-to-treat population are shown in Table S1.

**Table 2a:** Incidence of persistent HPV 16/18 and Vaccine Efficacy by Month 18 (mITT Cohort)

						95% Confidence Interval*		Statistical Comparisons***			
Arm	Enrolled (n)	HPV 16/18 naïve^ (mITT) (n)	Incident persistent HPV 16/18 (n)	Woman-years of Follow- up**	Incidence of persistent HPV 16/18 per 100 Woman-years	Lower Bound	Upper Bound	Comparison	Vaccine Efficacy	95% CI	P-value (Log-rank)
Nonavalent HPV	758	496	1	596.27	0.17	0.00	0.93	Nonavalent v. Meningococcal	97.5%	(81.7%, 99.7%)	<.0001
Bivalent HPV	760	489	1	589.38	0.17	0.00	0.95	Bivalent v. Meningococcal	97.5%	(81.6%, 99.7%)	<.0001
Meningococcal	757	473	36	527.35	6.83	4.78	9.45				

* Exact 95% confidence interval for incidence rate computed using the Poisson distribution.

** Follow-up time begins at 3 months and includes only women HPV 16/18 DNA-negative at month 0 and month 3, and antibody-negative at month 0.

*** Hazard ratios with 95% confidence intervals are estimated using a single Cox proportional hazards regression model with a three-way class variable for vaccine arm. The model is stratified by site, with Efron method for handling ties, and vaccine arm was the only covariate. Vaccine efficacy and 95% CI computed from the hazard ratio as 100*(1-HR). P-value (log-rank) computed for each comparison using the log-rank test.

^ HPV 16/18 naïve participants are those who tested negative for HPV 16/18 antibodies at enrollment and negative for HPV 16/18 DNA at enrollment and month three.

**Table 2b:** Incidence of persistent HPV 16/18/31/33/45/52/58 and Vaccine Efficacy by Month 18 (mITT Cohort)

						95% Confidence Interval*		Statistical Comparisons***			
Arm	Enrolled (n)	HPV 16/18/31/ 33/45/52/ 58 naïve^ (mITT) (n)	Incident persistent HPV 16/18/31/3 3/45/52/58 (n)	Woman- years of Follow- up**	Incidence of persistent HPV 16/18/31/33/4 5/52/58 per 100 Woman- years	Lower Bound	Upper Bound	Comparison	Vaccine Efficacy	95% CI	P-value (Log- rank)
Nonavalent HPV	758	325	4	389.18	1.03	0.28	2.63	Nonavalent v. Meningococcal	88.9%	(68.5%, 96.1%)	<.0001
Meningococcal	757	290	29	307.81	9.42	6.31	13.53				

* Exact 95% confidence interval for incidence rate computed using the Poisson distribution.

** Follow-up time amongst women HPV 16/18/31/33/45/52/58 DNA-negative at month 0 and month 3, and antibody-negative at month 0.

*** Hazard ratios with 95% confidence intervals are estimated using a single Cox proportional hazards regression model with a three-way class variable for vaccine arm. The model is stratified by site, with Efron method for handling ties, and vaccine arm was the only covariate. Vaccine efficacy and 95% CI computed from the hazard ratio as 100*(1-HR). P-value (log-rank) computed for each comparison using the log-rank test.

^ HPV 16/18/31/33/45/52/58 naïve participants are those who tested negative for HPV 16/18/31/33/45/52/58 antibodies at enrollment and negative for HPV 16/18/31/33/45/52/58 DNA at enrollment and month three.

**Table 3:** Participants experiencing adverse events (ITT)

	Randomized Arm			
	Nonavalent HPV	Bivalent HPV	Meningococcal	All

Enrolled, n	758	760	757	2275
Any SAE, n(%)	34 (4.5%)	39 (5.1%)	39 (5.2%)	112 (4.9%)
Any pregnancy related, n (%)	24 (3.2%)	19 (2.5%)	14 (1.8%)	57 (2.5%)
Any infection/inflammation, n (%)	9 (1.2%)	16 (2.1%)	21 (2.8%)	46 (2.0%)
Any injury, n (%)	0 (0.0%)	3 (0.4%)	4 (0.5%)	7 (0.3%)
Any mental health, n (%)	2 (0.3%)	1 (0.1%)	2 (0.3%)	5 (0.2%)

NOTE: Participants may have more than one event across, but not within, event type categories. SAE: Serious adverse event

## Data Availability

Data cannot be shared publicly because this study was conducted with approval from the Kenya Medical Research Institute (KEMRI) Scientific and Ethics Review Unit (SERU), which requires that data from studies (including de-identified data) are released only after SERU has provided written approval for additional analyses. A complete de-identified dataset sufficient to reproduce the study findings will be made available upon written request after approval from SERU. To request these data, please contact the KEN SHE Scientific Committee at icrc@uw.edu.
