# Supplementary material for: Efficacy of single-dose HPV vaccination among young African women
Source: NEJM Evid. Author manuscript. (PMC9172784; doi:10.1056/EVIDoa2100056)
Supplement: Supplement [file EVIDoa2100056_Supplement.pdf]

Table of Contents

KEN SHE STUDY TEAM 3

Table S1: Baseline characteristics of the ITT population 4

Table S2: Visit retention - ITT m18 analysis data 5

Table S3: Visit retention - HPV 16/18 mITT m18 analysis data 6

Table S4: Visit retention - HPV 16/18/31/33/45/52/58 mITT m18 analysis data 7

Table S5. Completeness of Endpoint Swab* Collection through Month 18 (HPV 16/18 mITT Cohort)....................8

Table S6. Number of Endpoint Swabs per Participant through Month 18 (HPV 16/18 mITT Cohort) 8

Table S7. Completeness of Endpoint Swab* Collection through Month 18 (HPV 16/18/31/33/45/52/58 mITT Cohort) 9

Table S8. Number of Endpoint Swabs per Participant through Month 18 (HPV 16/18/31/33/45/52/58 mITT Cohort) 9

Table S9. Incidence of persistent HPV 16/18 including visits beyond Month 18 (Extended Sensitivity Cohort) 10

Table S10. Incidence of persistent HPV 16/18/31/33/45/52/58 including visits beyond Month 18 (Extended Sensitivity Cohort) 11

Table S11. Incidence of persistent HPV 16/18 and Vaccine Efficacy by month 18 using Cervical Swabs Only (mITT Cohort) 12

Table S12. Incidence of persistent HPV 16/18/31/33/45/52/58 and Vaccine Efficacy by month 18 using Cervical Swabs Only (mITT Cohort) 13

Table S13. Summary of Tested Endpoint Swabs and Swab Collection Types 14

Table S14. Mean fluorescence intensity (MFI) cut-off values used for Luminex Antibody Assay of Enrollment Serum Specimens 15

Table S15a. Summary of mITT Endpoint HPV Types by Randomized Group (HPV 16/18 mITT Cohort)....................16

Table S15b. Summary of mITT Endpoint HPV Types by Randomized Group (HPV 16/18/31/33/45/52/58 mITT Cohort) 16

Table S16a. Summary of Follow-up Laboratory Results by Randomized Group (HPV 16/18 mITT Cohort) 17

Table S16b. Summary of Follow-up Laboratory Results by Randomized Group (HPV 16/18/31/33/45/52/58 mITT Cohort) 17

Table S17. Incidence of persistent HPV 16/18 and Vaccine Efficacy by Month 18 (ITT)....................18

Table S18. Incidence of persistent HPV 16/18/31/33/45/52/58 and Vaccine Efficacy by Month 18 (ITT)....................19

Table S19. Summary of Participants with only 1 post-Month 3 Swab Resulted for HPV DNA through Month 18 (mITT Cohorts)....................20

Table S20: Participants experiencing adverse events (ITT) 21

Table S21. Incidence of persistent HPV (Vaccine Types) by Month 18 (HPV type-specific mITT Cohorts)....................22

Table S22. Supplementary Table on the Representativeness of Study Participants....................23

Table S23. Incidence of persistent HPV 16/18 including visits beyond Month 18 (Sensitivity Cohort including participants with HPV antibodies at enrollment) 24
Table S24. Incidence of persistent HPV 16/18/31/33/45/52/58 including visits beyond Month 18 (Sensitivity Cohort including participants with HPV antibodies at enrollment)..25
Table S25. Exploratory analysis: Incidence of persistent HPV 31/33/45 (HPV 31/33/45 mITT cohort)..............26
Table S26. Exploratory analysis: Incidence of persistent HPV Types (Combined non-Vaccine Types, mITT sensitivity cohort)..27
Table S27: Baseline STI characteristics: modified intention-to-treat (mITT) cohort...28

KEN SHE STUDY TEAM

Listed in alphabetical order by institution.

Bill and Melinda Gates Foundation (Peter Dull, Caroline Wendell, Ruha Shadab); **Fred Hutchinson Cancer Research Center** (Elizabeth Brown, Deborah Donnell, Denise Galloway, Jody Carter, Leeya F. Pinder, Marci Wright, Priya R. Prabhu Robin Smith); **KEMRI Kisumu** (Elizabeth A. Bukusi, Maricianah Onono, Annette A. Opondo, Brian Mata, Byron I. Odhiambo, Catherine W. Mwakio, Charles O. Ochulo, Chrispine O. Abuya, Christine A. Olweny, Consolata A. Opondo, Cynthia Akinyi, David E. Muhoma, Debora A. Odhiambo, Dismas O. Congo, Donnavane A. Ondego, Florence A. Ondiek, Fyrose Makori, George O. Omondi, Gilbert C. Mutai, Hellen A. Olweyo, Imelda N. Imali, Imeldah N. Wakhungu, Irene A. Amada, Irine A. Odongo, Janet A. Okeyo, Joan A. Ongere, Job A. Ouma, Joseph O. Ayoo, Judith A. Osiro, Kevin O. Onyango, Kiplagat Kiptoo, Linet A. Okode, Lizzie N. Kabete, Lyna A. Memo, Maqline A. Achola, Maxwell A. Oluoch, Meldah O. Adipo, Mildred A. Owenga, Millicent A. Oronje, Moses O. Siaji, Nobert B. Walusala, Nollyne A. Okuku, Penina N. Amboka, Prudence A. Amolo, Rebecca A. Otieno, Reina Lenturkana, Renna A. Opere, Robai Mituyi, Sarah G. Obaje, Simon M. Muthusi, Thomas O. Odhul, Tobias O. Odwar, Veronica O. Atogo, Walter W. Otieno, Yuashita E. Hussein); **KEMRI Nairobi** (Betty Njoroge, Andrew Mutinda, Ann I. Namulen, Brenda Amollo, Chrispinus Wanyonyi, Edith W. Mwangi, Edna Nyandiga, Epines K. Chavangi, Esther K. Charles, Hellen W. Kimani, Irene Thuo, Jackline Muthoki, Milkah W. Wambui, Moses M. Mutinda, Peter Mogere, Priscillah Wangeci, Purity Kiwinda, Reginah N. Gichuki, Sahara H. Adan, Snaidah Ongachi, Syovata Kimanthi, Ted Opiyo, Umi W. Mugo, Veronica W. Muchoki, Victoria N. Kyengo, Victoria Oluoch, Vincent Juma); **KEMRI Thika** (Nelly Mugo, Anne Akinyi, Barbara Akinyi, Benedict Muchai, Caren Koli, Catherine Kiptinness, Charlene Biwott, Charles Mwangi, David Chege, Dennis Wafula, Diana Rubia, Edith Kimani, Edwin Mugo, Elizabeth Okwaro, Emily Anyango, Erick Koome, Faith Nyantika, Francis Khaemba, Fridah Nkatha, Gladys Namboka, Grace Muguro, Grace Ndung'u, Irene Njeru, Jacinta Nyokabi, Jelioth Muthoni, John Njoroge, Joshua Omari, Judith Achieng, Linet Makena, Lydiah Wambui, Lynda Oluoch, Margaret Mwangi, Margaret Wairima, Mary Kibatha, Mathew Irungu, Matilda Saina, Moses Musau, Nancy Kiarie, Nina Ouko, Peter Mogere, Peter Mwenda, Peterson Mwaniki, Praxides Pessah, Rosemary Ngacha, Sarah Mbaire, Scholastica Wanjiku, Snaidah Ongachi, Solomon Maina, Stephen Gakuo, Victoria Wambui, Vincent Juma, Zachary Gathu); **University of Washington, Mombasa** (R. Scott McClelland, Emmanuel Kabare, Fatma H. Mwidadi, Juma Shafi, Khamis Mwinyikai, Rukiya Hassan, Salwa Mustafa); **University of Washington, Seattle** (Ruanne Barnabas, Elizabeth Brown, Bobbi Nodell, Cara Bayer, Caroline H. Liou, Clare E. Brown, Connie Celum, Daphne Hamilton, Deborah Donnell, Deidra Montoya, Elena A. Rechkina, Elizabeth Harrington, Gui Lui, Hannah Leingang, Harald Haugen, Jared M. Baeten, Jasper Bleijs, Jack Knauer, Jenell C. Stewart, Jennifer Baugh, Jodi Greathouse, John Lin, Justice Quame-Amaglo, Kate B. Heller, Lara Kidoguchi, Meighan Krows, Rachel Johnson, Rachel L. Winer, Stephen L. Cherne, Susan Morrison, Toni Maddox, Torin Schaafsma); **DF/Net Research, Inc., Seattle** (Angela Williams, Bill Larson, Gavin Robertson, Krissa Gunderson, Lisa Ondrejcek, Rheanna Summers, Tadas Lukosevicius)

Contributions

RVB, DD, ERB, NM, and JMB designed the trial. KBH and TTS performed the data analyses. MO, RVB, NM and HL oversaw the operations of the trial. All authors contributed equally to results interpretation. RVB wrote the first draft of the manuscript. All authors contributed equally to the execution of the trial and critically reviewed and approved the finalized manuscript.

Table S1: Baseline characteristics of the ITT population

Characteristic	Category	Nonavalent HPV	Bivalent HPV	Meningococcal	All
	Total	758	760	757	2275
Age group (years)	15-17	453 (59.8%)	424 (55.8%)	424 (56.0%)	1301 (57.2%)
	18-20	305 (40.2%)	336 (44.2%)	333 (44.0%)	974 (42.8%)
Marital status	Never married	728 (96.0%)	713 (93.8%)	712 (94.1%)	2153 (94.6%)
	Married	25 (3.3%)	39 (5.1%)	32 (4.2%)	96 (4.2%)
	Previously Married	4 (0.5%)	6 (0.8%)	13 (1.7%)	23 (1.0%)
	Other	1 (0.1%)	2 (0.3%)	0 (0.0%)	3 (0.1%)
Education (highest level)	No schooling	1 (0.1%)	3 (0.4%)	3 (0.4%)	7 (0.3%)
	Primary school, some or complete	52 (6.9%)	47 (6.2%)	61 (8.1%)	160 (7.0%)
	Secondary school, some or complete	553 (73.0%)	551 (72.5%)	550 (72.7%)	1654 (72.7%)
	Post-secondary school	152 (20.1%)	159 (20.9%)	143 (18.9%)	454 (20.0%)
Earns an income of her own	No	665 (87.7%)	653 (85.9%)	656 (86.7%)	1974 (86.8%)
	Yes	93 (12.3%)	107 (14.1%)	101 (13.3%)	301 (13.2%)
Has a current main or steady sexual partner	No	209 (27.6%)	222 (29.2%)	211 (27.9%)	642 (28.2%)
	Yes	549 (72.4%)	538 (70.8%)	546 (72.1%)	1633 (71.8%)
Age when first had vaginal intercourse (years)	<15	190 (25.1%)	180 (23.7%)	170 (22.5%)	540 (23.7%)
	15-17	397 (52.4%)	415 (54.6%)	445 (58.8%)	1257 (55.3%)
	>=18	148 (19.5%)	155 (20.4%)	130 (17.2%)	433 (19.0%)
	Don't remember	23 (3.0%)	10 (1.3%)	12 (1.6%)	45 (2.0%)
Number of lifetime sexual partners	1	464 (61.2%)	484 (63.7%)	444 (58.7%)	1392 (61.2%)
	2	195 (25.7%)	176 (23.2%)	194 (25.6%)	565 (24.8%)
	>=3	99 (13.1%)	100 (13.2%)	119 (15.7%)	318 (14.0%)
Condom use with last vaginal sex	No	239 (31.5%)	244 (32.1%)	233 (30.8%)	716 (31.5%)
	Yes	358 (47.2%)	365 (48.0%)	367 (48.5%)	1090 (47.9%)
	No sex in past year	161 (21.2%)	151 (19.9%)	157 (20.7%)	469 (20.6%)
Syphilis	Negative	757 (99.9%)	760 (100.0%)	754 (99.6%)	2271 (99.8%)
	Positive	1 (0.1%)	0	1 (0.1%)	2 (0.1%)
	Not Done	0	0	2 (0.3%)	2 (0.1%)
C. trachomatis	Negative	665 (87.7%)	663 (87.2%)	651 (86.0%)	1979 (87.0%)
	Positive	93 (12.3%)	97 (12.8%)	106 (14.0%)	296 (13.0%)
N. gonorrhoeae	Negative	745 (98.3%)	738 (97.1%)	741 (97.9%)	2224 (97.8%)
	Positive	13 (1.7%)	22 (2.9%)	16 (2.1%)	51 (2.2%)
HSV-2	Negative	616 (81.3%)	597 (78.6%)	584 (77.1%)	1797 (79.0%)
	Positive	141 (18.6%)	162 (21.3%)	173 (22.9%)	476 (20.9%)
	Indeterminate	1 (0.1%)	1 (0.1%)	0	2 (0.1%)
Bacterial vaginosis*	Negative	604 (79.7%)	576 (75.8%)	587 (77.5%)	1767 (77.7%)
	Positive	154 (20.3%)	183 (24.1%)	170 (22.5%)	507 (22.3%)
	Not Done	0	1 (0.1%)	0	1 (0.0%)
Trichomonas vaginalis	Negative	723 (95.4%)	728 (95.8%)	722 (95.4%)	2173 (95.5%)
	Positive	35 (4.6%)	32 (4.2%)	35 (4.6%)	102 (4.5%)

*Nugent scores 7-10 were classified as BV positive and Nugent score 0-6 were classified as BV negative.

Table S2: Visit retention - ITT m18 analysis data

		Month 3	Month 6	Month 9	Month 12	Month 15	Month 18
**Randomized Group**							
Nonavalent HPV	Expected	758	758	758	758	758	742
	Completed	710 (93.7%)	651 (85.9%)	723 (95.4%)	642 (84.7%)	738 (97.4%)	688 (92.7%)
	Missed	48 (6.3%)	107 (14.1%)	35 (4.6%)	116 (15.3%)	20 (2.6%)	54 (7.3%)
Bivalent HPV	Expected	760	760	760	760	760	746
	Completed	712 (93.7%)	644 (84.7%)	731 (96.2%)	662 (87.1%)	742 (97.6%)	701 (94.0%)
	Missed	48 (6.3%)	116 (15.3%)	29 (3.8%)	98 (12.9%)	18 (2.4%)	45 (6.0%)
Meningococcal	Expected	757	756	756	756	756	742
	Completed	699 (92.3%)	637 (84.3%)	719 (95.1%)	650 (86.0%)	735 (97.2%)	684 (92.2%)
	Missed	58 (7.7%)	119 (15.7%)	37 (4.9%)	106 (14.0%)	21 (2.8%)	58 (7.8%)
All	Expected	2275	2274	2274	2274	2274	2230
	Completed	2121 (93.2%)	1932 (85.0%)	2173 (95.6%)	1954 (85.9%)	2215 (97.4%)	2073 (93.0%)
	Missed	154 (6.8%)	342 (15.0%)	101 (4.4%)	320 (14.1%)	59 (2.6%)	157 (7.0%)

NOTE: A visit is counted as expected if a participant has completed the visit within the expected window, or once the participant's visit window has closed. A visit is counted as missed only once the last visit window has closed.

Table S3: Visit retention - HPV 16/18 mITT m18 analysis data

Randomized Group		Month 3	Month 6	Month 9	Month 12	Month 15	Month 18
Nonavalent HPV	Expected	496	496	496	496	496	489
	Completed	496 (100.0%)	454 (91.5%)	481 (97.0%)	436 (87.9%)	489 (98.6%)	460 (94.1%)
	Missed	0	42 (8.5%)	15 (3.0%)	60 (12.1%)	7 (1.4%)	29 (5.9%)
Bivalent HPV	Expected	489	489	489	489	489	486
	Completed	489 (100.0%)	437 (89.4%)	477 (97.5%)	436 (89.2%)	480 (98.2%)	461 (94.9%)
	Missed	0	52 (10.6%)	12 (2.5%)	53 (10.8%)	9 (1.8%)	25 (5.1%)
Meningococcal	Expected	473	473	473	473	473	468
	Completed	473 (100.0%)	422 (89.2%)	455 (96.2%)	419 (88.6%)	464 (98.1%)	434 (92.7%)
	Missed	0	51 (10.8%)	18 (3.8%)	54 (11.4%)	9 (1.9%)	34 (7.3%)
All	Expected	1458	1458	1458	1458	1458	1443
	Completed	1458 (100.0%)	1313 (90.1%)	1413 (96.9%)	1291 (88.5%)	1433 (98.3%)	1355 (93.9%)
	Missed	0	145 (9.9%)	45 (3.1%)	167 (11.5%)	25 (1.7%)	88 (6.1%)

NOTE: A visit is counted as expected if a participant has completed the visit within the expected window, or once the visit window has closed. A visit is counted as missed only once the visit window has closed.

Table S4: Visit retention - HPV 16/18/31/33/45/52/58 mITT m18 analysis data

		Month 3	Month 6	Month 9	Month 12	Month 15	Month 18
**Randomized Group**							
Nonavalent HPV	Expected	325	325	325	325	325	321
	Completed	325 (100.0%)	300 (92.3%)	316 (97.2%)	291 (89.5%)	322 (99.1%)	306 (95.3%)
	Missed	0	25 (7.7%)	9 (2.8%)	34 (10.5%)	3 (0.9%)	15 (4.7%)
Meningococcal	Expected	290	290	290	290	290	286
	Completed	290 (100.0%)	262 (90.3%)	280 (96.6%)	255 (87.9%)	283 (97.6%)	267 (93.4%)
	Missed	0	28 (9.7%)	10 (3.4%)	35 (12.1%)	7 (2.4%)	19 (6.6%)
All	Expected	615	615	615	615	615	607
	Completed	615 (100.0%)	562 (91.4%)	596 (96.9%)	546 (88.8%)	605 (98.4%)	573 (94.4%)
	Missed	0	53 (8.6%)	19 (3.1%)	69 (11.2%)	10 (1.6%)	34 (5.6%)

NOTE: A visit is counted as expected if a participant has completed the visit within the expected window, or once the visit window has closed. A visit is counted as missed only once the visit window has closed.

Table S5. Completeness of Endpoint Swab* Collection through Month 18 (HPV 16/18 mITT Cohort)

	Swab 1 (Month 6)		Swab 2 (Month 12)		Swab 3 (Month 18)	
**Randomized Group**	**Expected (n)****	**Completed, n (%)**	**Expected (n)****	**Completed, n (%)**	**Expected (n)****	**Completed, n (%)**
Nonavalent HPV	496	496 (100.0%)	496	485 (97.8%)	463	434 (93.7%)
Bivalent HPV	489	489 (100.0%)	489	479 (98.0%)	467	445 (95.3%)
Meningococcal	473	472 (99.8%)	473	466 (98.5%)	445	415 (93.3%)
All	1458	1457 (99.9%)	1458	1430 (98.1%)	1375	1294 (94.1%)

*Endpoint swabs defined as post-Month 3 cervical vaginal or self-collected vaginal swabs at least 4 months apart. Three collected endpoint swabs were not resulted for HPV DNA,
**Month 6 and Month 12 swabs are counted as expected if collected, or if the corresponding visit windows have closed. Month 18 swabs are counted as expected if collected or if >8.5 months have passed since the previous swab collection.

Table S6. Number of Endpoint Swabs per Participant through Month 18 (HPV 16/18 mITT Cohort)

	Number of Endpoint Swabs per Participant*									
	0		1		2		3		Total	
**Randomized Group**	**n**	**Row %**	**n**	**Row %**	**n**	**Row %**	**n**	**Row %**	**n**	**%**
Nonavalent HPV	0	0.0%	11	2.2%	51	10.3%	434	87.5%	496	34.0%
Bivalent HPV	0	0.0%	10	2.0%	34	7.0%	445	91.0%	489	33.5%
Meningococcal	1	0.2%	6	1.3%	51	10.8%	415	87.7%	473	32.4%
	1	0.1%	27	1.9%	136	9.3%	1294	88.8%	1458	100.0%

*Endpoint swabs defined as post-Month 3 cervical vaginal or self-collected vaginal swabs at least 4 months apart. Three collected endpoint swabs were not resulted for HPV DNA.

Table S7. Completeness of Endpoint Swab* Collection through Month 18 (HPV 16/18/31/33/45/52/58 mITT Cohort)

	Swab 1 (Month 6)		Swab 2 (Month 12)		Swab 3 (Month 18)	
**Randomized Group**	**Expected (n)****	**Completed, n (%)**	**Expected (n)****	**Completed, n (%)**	**Expected (n)****	**Completed, n (%)**
Nonavalent HPV	325	325 (100.0%)	325	319 (98.2%)	309	293 (94.8%)
Meningococcal	290	289 (99.7%)	290	285 (98.3%)	273	253 (92.7%)
All	615	614 (99.8%)	615	604 (98.2%)	582	546 (93.8%)

*Endpoint swabs defined as post-Month 3 cervical vaginal or self-collected vaginal swabs at least 4 months apart. Two collected endpoint swabs were not resulted for HPV DNA.
**Month 6 and Month 12 swabs are counted as expected if collected, or if the corresponding visit windows have closed. Month 18 swabs are counted as expected if collected or if >8.5 months have passed since the previous swab collection.

Table S8. Number of Endpoint Swabs per Participant through Month 18 (HPV 16/18/31/33/45/52/58 mITT Cohort)

	Number of Endpoint Swabs per Participant*									
	0		1		2		3		Total	
**Randomized Group**	**n**	**Row %**	**n**	**Row %**	**n**	**Row %**	**n**	**Row %**	**n**	**%**
Nonavalent HPV	0	0.0%	6	1.8%	26	8.0%	293	90.2%	325	52.8%
Meningococcal	1	0.3%	4	1.4%	32	11.0%	253	87.2%	290	47.2%
	1	0.2%	10	1.6%	58	9.4%	546	88.8%	615	100.0%

*Endpoint swabs defined as post-Month 3 cervical vaginal or self-collected vaginal swabs at least 4 months apart. Two collected endpoint swabs were not resulted for HPV DNA.

Table S9. Incidence of persistent HPV 16/18 including visits beyond Month 18 (Extended Sensitivity Cohort)

Group	Enrolled (n)	HPV 16/18 naïve (mITT extended sensitivity) (n)	Incident persistent HPV 16/18 (n)	Woman-years of Follow-up**	Incidence of persistent HPV 16/18 per 100 Woman-years	95% Confidence Interval*: Lower Bound	95% Confidence Interval*: Upper Bound	Statistical Comparisons***: Comparison	Statistical Comparisons***: Vaccine Efficacy
Nonavalent	758	429	0	499.67	0.00	0.00	0.74	Nonavalent v. Meningococcal	100.0%
Bivalent	760	404	0	470.32	0.00	0.00	0.78	Bivalent v. Meningococcal	100.0%
Meningococcal	757	380	16	410.53	3.90	2.23	6.33		

*Exact 95% confidence interval for incidence rate computed using the Poisson distribution.

**Follow-up time amongst women HPV 16/18 DNA-negative at month 0, month 3, and month 6, and antibody-negative at month 0.

***Vaccine efficacy computed as 100*(1-Crude Incidence Rate Ratio).

No multiplicity adjustments for the secondary and exploratory end points were defined in our Statistical Analysis Plan. Therefore, only point estimates and 95% confidence intervals are provided. The confidence intervals have not been adjusted for multiple comparisons and should not be used to infer definitive treatment effects.

Table S10. Incidence of persistent HPV 16/18/31/33/45/52/58 including visits beyond Month 18 (Extended Sensitivity Cohort)

						95% Confidence Interval*		Statistical Comparisons***		
**Group**	**Enrolled (n)**	**HPV 16/ 18/31/33/ 45/52/58 naïve (mITT extended sensitivity) (n)**	**Incident persistent HPV 16 18 31 33 45 52 58 (n)**	**Woman-years of Follow-up****	**Incidence of persistent HPV 16 18 31 33 45 52 58 per 100 Woman-years**	**Lower Bound**	**Upper Bound**	**Comparison**	**Vaccine Efficacy**	**95% CI**
Nonavalent	758	264	1	308.72	0.32	0.01	1.81	Nonavalent v. Meningococcal	95.02%	(62.14%, 99.35%)
Meningococcal	757	210	14	220.17	6.36	3.48	10.67			

*Exact 95% confidence interval for incidence rate computed using the Poisson distribution.

**Follow-up time amongst women HPV 16/18/31/33/45/52/58 DNA-negative at month 0, month 3 and month 6, and antibody-negative at month 0.

***"*Hazard ratios with 95% confidence intervals are estimated using a single Cox proportional hazards regression model with a three-way class variable for vaccine group. The model is stratified by site, with Efron method for handling ties, and vaccine arm was the only covariate. Vaccine efficacy and 95% CI computed from the hazard ratio as [100*(1-HR)].

No multiplicity adjustments for the secondary and exploratory end points were defined in our Statistical Analysis Plan. Therefore, only point estimates and 95% confidence intervals are provided. The confidence intervals have not been adjusted for multiple comparisons and should not be used to infer definitive treatment effects.

Table S11. Incidence of persistent HPV 16/18 and Vaccine Efficacy by month 18 using Cervical Swabs Only (mITT Cohort)

						95% Confidence Interval*		Statistical Comparisons***		
Group	Enrolled (n)	HPV 16/18 naïve (mITT) (n)	Incident persistent HPV 16/18 (n)	Woman-years of Follow-up**	Incidence of persistent HPV 16/18 per 100 Woman-years	Lower Bound	Upper Bound	Comparison	Vaccine Efficacy	95% CI
Nonavalent HPV	758	496	1	568.96	0.18	0.00	0.98	Nonavalent v. Meningococcal	97.27%	(80.01%, 99.63%)
Bivalent HPV	760	489	1	563.14	0.18	0.00	0.99	Bivalent v. Meningococcal	97.26%	(79.96%, 99.63%)
Meningococcal	757	473	33	504.44	6.54	4.50	9.19			

*Exact 95% confidence interval for incidence rate computed using the Poisson distribution.";
**Follow-up time amongst women HPV 16/18 DNA-negative at month 0 and month 3, and antibody-negative at month 0.
***Hazard ratios with 95% confidence intervals are estimated using a single Cox proportional hazards regression model with a three-way class variable for vaccine arm. The model is stratified by site, with Efron method for handling ties, and vaccine group was the only covariate. Vaccine efficacy and 95% CI computed from the hazard ratio as 100*(1-HR).
No multiplicity adjustments for the secondary and exploratory end points were defined in our Statistical Analysis Plan. Therefore, only point estimates and 95% confidence intervals are provided. The confidence intervals have not been adjusted for multiple comparisons and should not be used to infer definitive treatment effects.

Table S12. Incidence of persistent HPV 16/18/31/33/45/52/58 and Vaccine Efficacy by month 18 using Cervical Swabs Only (mITT Cohort)

						95% Confidence Interval*		Statistical Comparisons***		
Group	Enrolled (n)	HPV 16/18/31/33/45/52/58 naïve (mITT) (n)	Incident persistent HPV 16/18/31/33/45/52/58 (n)	Woman-years of Follow-up**	Incidence of persistent HPV 16/18/31/33/45/52/58 per 100 Woman-years	Lower Bound	Upper Bound	Comparison	Vaccine Efficacy	95% CI
Nonavalent HPV	758	325	3	370.47	0.81	0.17	2.37	Nonavalent v. Meningococcal	91.42%	(71.78%, 97.39%)
Meningococcal	757	290	28	292.49	9.57	6.36	13.84			

*Exact 95% confidence interval for incidence rate computed using the Poisson distribution.

**Follow-up time amongst women HPV 16/18/31/33/45/52/58 DNA-negative at month 0 and month 3, and antibody-negative at month 0.

***Hazard ratios with 95% confidence intervals are estimated using a single Cox proportional hazards regression model with a three-way class variable for vaccine group. The model is stratified by site, with Efron method for handling ties, and vaccine group was the only covariate. Vaccine efficacy and 95% CI computed from the hazard ratio as [100*(1-HR)].

No multiplicity adjustments for the secondary and exploratory end points were defined in our Statistical Analysis Plan. Therefore, only point estimates and 95% confidence intervals are provided. The confidence intervals have not been adjusted for multiple comparisons and should not be used to infer definitive treatment effects.

Table S13. Summary of Tested Endpoint Swabs and Swab Collection Types

Analysis Cohort	Total Endpoint Swabs (n)	Cervical Swabs (n)	%	Self-Collected Swabs (n)	%
ITT	6402	6042	94.38%	360	5.62%
HPV 16/18 mITT	4178	3982	95.31%	196	4.69%
HPV 16/18/31/33/45/52/58 mITT	1762	1689	95.86%	73	4.14%

NOTE: Endpoint swabs defined as post-Month 3 cervical or self-collected vaginal swabs at least 4 months apart.

Table S14. Mean fluorescence intensity (MFI) cut-off values used for Luminex Antibody Assay of Enrollment Serum Specimens

	MFI cut-off Values*	
	Luminex HPV Antibody Assay Date	
	July 2019-January 2021	Feb 2021-July 2021
HPV 16	1500	1000
HPV 18	1000	500
HPV 31	1500	1000
HPV 33	1500	500
HPV 45	1500	500
HPV 52	2500	1000
HPV 58	1500	1000

*Enrollment serum specimens with MFI values greater than or equal the pre-defined cut-offs above were considered positive antibody results.

Table S15a. Summary of mITT Endpoint HPV Types by Randomized Group (HPV 16/18 mITT Cohort)

	Randomized Group			
	Nonavalent HPV	Bivalent HPV	Meningococcal	Total
HPV Type*	n	n	n	n
16	1	1	28	30
16,18	0	0	1	1
18	0	0	7	7
Total	1	1	36	38

*Vaccine-specific HPV type(s) detected at two consecutive visits at least four months apart. If two or more HPV types are listed, both/all types were detected at the consecutive visits.

Table S15b. Summary of mITT Endpoint HPV Types by Randomized Group (HPV 16/18/31/33/45/52/58 mITT Cohort)

	Randomized Group			
	Nonavalent HPV	Bivalent HPV	Meningococcal	Total
HPV Type*	n	n	n	n
16	1	0	12	13
16,52	0	0	1	1
16,52,58	0	0	1	1
18	0	0	4	4
31	1	5	1	7
31,58	0	1	0	1
33	0	1	1	2
45	0	2	1	3
45,52	0	0	1	1
52	0	9	6	15
58	2	11	1	14
Total	4	29	29	62

*Vaccine-specific HPV type(s) detected at two consecutive visits at least four months apart. If two or more HPV types are listed, both/all types were detected at the consecutive visits.

Table S16a. Summary of Follow-up Laboratory Results by Randomized Group (HPV 16/18 mITT Cohort)

		C. trachomatis			N. gonorrhoeae		
**Randomized Group**	**Enrolled (n)**	**Positive test results for chlamydia (n)***	**Participants with one or more positive test results for chlamydia (n)**	**%**	**Positive test results for gonorrhea (n)***	**Participants with one or more positive test results for gonorrhea (n)**	**%**
Nonavalent HPV	496	121	99	20.0%	43	37	7.5%
Bivalent HPV	489	131	113	23.1%	37	33	6.7%
Meningococcal	473	107	89	18.8%	34	31	6.6%
All	1458	359	301	20.6%	114	101	6.9%

*Includes testing at six monthly follow-up visits completed within visit window with non-missing HPV DNA swabs.

Table S16b. Summary of Follow-up Laboratory Results by Randomized Group (HPV 16/18/31/33/45/52/58 mITT Cohort)

		C. trachomatis			N. gonorrhoeae		
**Randomized Group**	**Enrolled (n)**	**Positive test results for chlamydia (n)***	**Participants with one or more positive test results for chlamydia (n)**	**%**	**Positive test results for gonorrhea (n)***	**Participants with one or more positive test results for gonorrhea (n)**	**%**
Nonavalent HPV	325	88	73	22.5%	26	23	7.1%
Meningococcal	290	58	49	16.9%	17	16	5.5%
All	615	146	122	19.8%	43	39	6.3%

*Includes testing at six monthly follow-up visits completed within visit window with non-missing HPV DNA swabs.

Table S17. Incidence of persistent HPV 16/18 and Vaccine Efficacy by Month 18 (ITT)

					95% Confidence Interval*		Statistical Comparisons***		
**Arm**	**Enrolled (n)**	**Incident persistent HPV 16/18 (n)**	**Woman-years of Follow-up****	**Incidence of persistent HPV 16/18 per 100 Woman-years**	**Lower Bound**	**Upper Bound**	**Comparison**	**Vaccine Efficacy**	**95% CI**
Nonavalent	758	38	1061.00	3.58	2.53	4.92	Nonavalent v. Meningococcal	62.32%	(45.13%, 74.12%)
Bivalent	760	34	1067.75	3.18	2.21	4.45	Bivalent v. Meningococcal	66.55%	(50.54%, 77.38%)
Meningococcal	757	96	994.90	9.65	7.82	11.78			

*Exact 95% confidence interval for incidence rate computed using the Poisson distribution.

**Follow-up time amongst randomized women.

***Hazard ratios with 95% confidence intervals are estimated using a single Cox proportional hazards regression model with a three-way class variable for vaccine arm. The model is stratified by site, with Efron method for handling ties, and vaccine arm was the only covariate. Vaccine efficacy and 95% CI computed from the hazard ratio as 100*(1-HR).

No multiplicity adjustments for the secondary and exploratory end points were defined in our Statistical Analysis Plan. Therefore, only point estimates and 95% confidence intervals are provided. The confidence intervals have not been adjusted for multiple comparisons and should not be used to infer definitive treatment effects.

Table S18. Incidence of persistent HPV 16/18/31/33/45/52/58 and Vaccine Efficacy by Month 18 (ITT)

					95% Confidence Interval*		Statistical Comparisons***		
**Arm**	**Enrolled (n)**	**Incident persistent HPV 16/18/ 31/33/45/52/58 (n)**	**Woman-years of Follow-up****	**Incidence of persistent HPV 16/18/31/33/45/52/58 per 100 Woman-years**	**Lower Bound**	**Upper Bound**	**Comparison**	**Vaccine Efficacy**	**95% CI**
Nonavalent	758	98	1002.25	9.78	7.94	11.92	Nonavalent v. Meningococcal	51.86%	(38.50%, 62.31%)
Bivalent	760	168	923.00	18.20	15.55	21.17			
Meningococcal	757	186	902.34	20.61	17.76	23.80			

*Exact 95% confidence interval for incidence rate computed using the Poisson distribution.

**Follow-up time amongst randomized women.

***Hazard ratios with 95% confidence intervals are estimated using a single Cox proportional hazards regression model with a three-way class variable for vaccine arm. The model is stratified by site, with Efron method for handling ties, and vaccine arm was the only covariate. Vaccine efficacy and 95% CI computed from the hazard ratio as [100*(1-HR)].

No multiplicity adjustments for the secondary and exploratory end points were defined in our Statistical Analysis Plan. Therefore, only point estimates and 95% confidence intervals are provided. The confidence intervals have not been adjusted for multiple comparisons and should not be used to infer definitive treatment effects.

Table S19. Summary of Participants with only 1 post-Month 3 Swab Resulted for HPV DNA through Month 18 (mITT Cohorts)

mITT Cohort	N	Only 1 post-month 3 Swab with HPV DNA Results (n)	Positive (n)*	%
HPV 16/18	1458	26	0	0.0%
HPV 16/18/31/33/45/52/58	615	10	0	0.0%

*Positive result for respective HPV types (for HPV 16/18 in the HPV 16/18 cohort, or HPV 16/18/31/33/45/52/58 in the HPV 16/18/31/33/45/52/58 cohort.

Table S20: Participants experiencing adverse events (ITT)

	Randomized Arm				P-value*
	Nonavalent HPV	Bivalent HPV	Meningococcal	All	
Enrolled, n	758	760	757	2275	
Any SAE, n(%)	34 (4.5%)	39 (5.1%)	39 (5.2%)	112 (4.9%)	0.8053
Any pregnancy related, n (%)	24 (3.2%)	19 (2.5%)	14 (1.8%)	57 (2.5%)	0.2744
Any infection/inflammation, n (%)	9 (1.2%)	16 (2.1%)	21 (2.8%)	46 (2.0%)	0.0775
Any injury, n (%)	0 (0.0%)	3 (0.4%)	4 (0.5%)	7 (0.3%)	0.7524
Any mental health, n (%)	2 (0.3%)	1 (0.1%)	2 (0.3%)	5 (0.2%)	0.1253

*P-value computed using Fisher's exact test.

NOTE: Participants may have more than one event across, but not within, event type categories. SAE: Serious adverse event

Table S21. Incidence of persistent HPV (Vaccine Types) by Month 18 (HPV type-specific mITT Cohorts)

							95% Confidence Interval*	
HPV Type	Arm	Enrolled (n)	HPV type-naive (mITT) (n)	Incident persistent HPV (n)	Woman-years of Follow-up**	Incidence of persistent HPV per 100 Woman-years	Lower Bound	Upper Bound
16	Nonavalent	758	521	1	626.53	0.16	0.00	0.89
	Bivalent	760	528	1	636.24	0.16	0.00	0.88
	Meningococcal	757	502	32	565.89	5.65	3.87	7.98
	All	2275	1551	34	1828.66	1.86	1.29	2.60
18	Nonavalent	758	652	0	785.97	0.00	0.00	0.47
	Bivalent	760	634	0	763.38	0.00	0.00	0.48
	Meningococcal	757	622	12	732.67	1.64	0.85	2.86
	All	2275	1908	12	2282.02	0.53	0.27	0.92
31	Nonavalent	758	622	1	748.62	0.13	0.00	0.74
	Bivalent	760	619	12	734.21	1.63	0.85	2.86
	Meningococcal	757	595	5	704.36	0.71	0.23	1.66
	All	2275	1836	18	2187.19	0.82	0.49	1.30
33	Nonavalent	758	695	0	838.96	0.00	0.00	0.44
	Bivalent	760	690	3	829.78	0.36	0.08	1.06
	Meningococcal	757	679	3	809.96	0.37	0.08	1.08
	All	2275	2064	6	2478.70	0.24	0.09	0.53
45	Nonavalent	758	605	2	727.83	0.27	0.03	0.99
	Bivalent	760	599	9	711.46	1.26	0.58	2.40
	Meningococcal	757	596	4	703.36	0.57	0.16	1.46
	All	2275	1800	15	2142.65	0.70	0.39	1.16
52	Nonavalent	758	590	1	710.76	0.14	0.00	0.78
	Bivalent	760	586	22	676.16	3.25	2.04	4.93
	Meningococcal	757	557	26	643.58	4.04	2.64	5.92
	All	2275	1733	49	2030.51	2.41	1.79	3.19
58	Nonavalent	758	573	5	689.53	0.73	0.24	1.69
	Bivalent	760	568	24	659.18	3.64	2.33	5.42
	Meningococcal	757	563	12	657.03	1.83	0.94	3.19
	All	2275	1704	41	2005.74	2.04	1.47	2.77

*Exact 95% confidence interval for incidence rate computed using the Poisson distribution.
**Follow-up time amongst women HPV-type DNA negative at month 0 and month 3 and HPV-type antibody negative at month 0

Table S22. Supplementary Table on the Representativeness of Study Participants

Category	HPV
Condition under investigation	Persistent infection with human papillomavirus (HPV). While most of these infections resolve without intervention, some infections persist and progress to dysplastic lesion. Thus, persistent HPV infection is used as a surrogate marker for precancerous lesions.
Special considerations related to:	
Sex and gender	Persistent cervical HPV infections occur in females. The study was limited to participants with female sex.
Age	HPV prevalence increases with age after sexual debut. In an HPV prevalence study in Kenya, HPV prevalence was 21.3% among 498 women age 18-74 years, with HPV prevalence decreasing with age. HPV prevalence was 28% among women younger than 30 years.^
Race or ethnic group	Cervical cancer affects Black persons disproportionately in South Africa.*
Geography	There are marked disparities in cervical cancer incidence globally. Almost 90% of the more than 600,000 new cervical cancer cases and 340,000 cervical cancer deaths in 2020 occurred in low- and middle-income countries (LMICs).
Other considerations	Trial eligibility criteria included one or more lifetime sexual partners.
Overall representativeness of this trial	The study did not enroll participant who were not sexually active and thus are participants do not represent the general population of 15–20-year-old adolescents and young women in Kenya. The participants in the clinical trial demonstrated the expected prevalence of HPV among young women in Kenya after sexual debut (29% in the clinical trial compared with 28% in a prevalence study in Kenya). Biological sex was reported by the participants prior to enrollment as enrollment procedures included cervical swabs. The prevalence of HPV among young women seen in this study is comparable to HPV prevalence in the United States: HPV prevalence was 24.5% (95% CI, 19.6%-30.5%) among females aged 14 to 19 years in a US national survey. All participants in the study were Black African, as is appropriate for the setting of a clinical trial in Kenya. The clinical trial population is comparable with sexually active 15–20-year-olds in Kenya and other settings.

^Ngugi C.W.1, Schmidt D, Wanyoro RK, Boga H, Wanzala P5, Muigai A.W.T, Mbithi J.N, von Knebel, Doeberitz M, Reuschenbach M. Prevalence of Human Papillomavirus infection by age and cervical cytology in Thika, Kenya. Afr J Health Sci. 2011; 19:52-62. *Bailie RS, Selvey CE, Bourne D, Bradshaw D. Trends in cervical cancer mortality in South Africa. Int J Epidemiol. 1996 Jun;25(3):488-93. doi: 10.1093/ije/25.3.488. PMID: 8671548. [+]Dunne EF, Unger ER, Sternberg M, et al. Prevalence of HPV Infection Among Females in the United States. JAMA. 2007;297(8):813–819. doi:10.1001/jama.297.8.813

Table S23. Incidence of persistent HPV 16/18 including visits beyond Month 18 (Sensitivity Cohort including participants with HPV antibodies at enrollment)

						95% Confidence Interval*		Statistical Comparisons***		
Arm	Enrolled (n)	HPV 16/18 naïve at baseline (mITT sensitivity) (n)	Incident persistent HPV 16/18 (n)	Woman-years of Follow-up**	Incidence of persistent HPV 16/18 per 100 Woman-years	Lower Bound	Upper Bound	Comparison	Vaccine Efficacy	95% CI
Nonavalent	758	569	1	798.26	0.13	0.00	0.70	Nonavalent v. Meningococcal	98.15%	(86.63%, 99.74%)
Bivalent	760	561	3	792.99	0.38	0.08	1.11	Bivalent v. Meningococcal	94.43%	(82.12%, 98.27%)
Meningococcal	757	543	48	694.08	6.92	5.10	9.17			

*Exact 95% confidence interval for incidence rate computed using the Poisson distribution.

**Follow-up time amongst women HPV 16/18 DNA-naïve at month 0 and month 3 (antibody results not used).

***Hazard ratios with 95% confidence intervals are estimated using a single Cox proportional hazards regression model with a three-way class variable for vaccine arm. The model is stratified by site, with Efron method for handling ties, and vaccine arm was the only covariate. Vaccine efficacy and 95% CI computed from the hazard ratio as 100*(1-HR).

No multiplicity adjustments for the secondary and exploratory end points were defined in our Statistical Analysis Plan. Therefore, only point estimates and 95% confidence intervals are provided. The confidence intervals have not been adjusted for multiple comparisons and should not be used to infer definitive treatment effects.

Table S24. Incidence of persistent HPV 16/18/31/33/45/52/58 including visits beyond Month 18 (Sensitivity Cohort including participants with HPV antibodies at enrollment)

						95% Confidence Interval*		Statistical Comparisons***		
Arm	Enrolled (n)	HPV 16/18/31 33/45/52 /58 naïve at baseline (mITT sensitivity) (n)	Incident persistent HPV 16/18/ 31/33/45/52/58 (n)	Woman-years of Follow-up**	Incidence of persistent HPV 16/18/31/33/45/52/58 per 100 Woman-years	Lower Bound	Upper Bound	Comparison	Vaccine Efficacy	95% CI
Nonavalent	758	437	7	601.86	1.16	0.47	2.40	Nonavalent v. Meningococcal	89.28%	(76.39%, 95.13%)
Meningococcal	757	392	52	474.81	10.95	8.18	14.36			

*Exact 95% confidence interval for incidence rate computed using the Poisson distribution.

**Follow-up time amongst women HPV 16/18/31/33/45/52/58 DNA-naïve at month 0 and month 3 (antibody results not used).

***Hazard ratios with 95% confidence intervals are estimated using a single Cox proportional hazards regression model with a three-way class variable for vaccine arm. The model is stratified by site, with Efron method for handling ties, and vaccine arm was the only covariate. Vaccine efficacy and 95% CI computed from the hazard ratio as [100*(1-HR)].

No multiplicity adjustments for the secondary and exploratory end points were defined in our Statistical Analysis Plan. Therefore, only point estimates and 95% confidence intervals are provided. The confidence intervals have not been adjusted for multiple comparisons and should not be used to infer definitive treatment effects.

Table S25. Exploratory analysis: Incidence of persistent HPV 31/33/45 (HPV 31/33/45 mITT cohort)

						95% Confidence Interval*		Statistical Comparisons***		
**Arm**	**Enrolled (n)**	**HPV 31/33/45 naive (mITT) (n)**	**Incident persistent HPV 31/33/45 (n)**	**Woman-years of Follow-up****	**Incidence of persistent HPV 31/33/45 per 100 Woman-years**	**Lower Bound**	**Upper Bound**	**Comparison**	**Vaccine Efficacy**	**95% CI**
Nonavalent	758	530	3	636.21	0.47	0.10	1.38	Nonavalent v. Meningococcal	69.31%	(-13.33%, 91.69%)
Bivalent	760	516	21	600.58	3.50	2.16	5.35	Bivalent v. Meningococcal	-129.7%	(-401.7%, -5.20%)
Meningococcal	757	506	9	586.36	1.53	0.70	2.91			

*Exact 95% confidence interval for incidence rate computed using the Poisson distribution.

**Follow-up time amongst women HPV 31/33/45 DNA-naïve at month 0 and month 3, and antibody-negative at month 0.

***Hazard ratios with 95% confidence intervals are estimated using a single Cox proportional hazards regression model with a three-way class variable for vaccine arm. The model is stratified by site, with Efron method for handling ties, and vaccine arm was the only covariate. Vaccine efficacy and 95% CI computed from the hazard ratio as [100*(1-HR)].

No multiplicity adjustments for the secondary and exploratory end points were defined in our Statistical Analysis Plan. Therefore, only point estimates and 95% confidence intervals are provided. The confidence intervals have not been adjusted for multiple comparisons and should not be used to infer definitive treatment effects.

Table S26. Exploratory analysis: Incidence of persistent HPV Types (Combined non-Vaccine Types, mITT sensitivity cohort)

						95% Confidence Interval*	
Arm	Enrolled (n)	HPV non-Vaccine Type-naïve (mITT) (n)	Incident persistent HPV (n)	Woman-years of Follow-up**	Incidence of persistent HPV per 100 Woman-years	Lower Bound	Upper Bound
Nonavalent	758	247	53	238.90	22.19	16.62	29.02
Bivalent	760	241	55	224.40	24.51	18.46	31.90
Meningococcal	757	250	53	234.08	22.64	16.96	29.62
All	2275	738	161	697.38	23.09	19.66	26.94

*Exact 95% confidence interval for incidence rate computed using the Poisson distribution.
**Follow-up time amongst women non-vaccine HPV-type DNA negative at month 0 and month 3 (women are excluded if HPV DNA positive at month 0 or month 3 for any of HPV 26/35/39/40/42/43/44/51/53/54/56/59/60/61/66/68/70/73/82)

Table S27: Baseline STI characteristics: modified intention-to-treat (mITT) cohort

		HPV 16/18 mITT			HPV 16/18/31/33/45/52/58 mITT	
		**Nonavalent HPV**	**Bivalent HPV**	**Meningococcal**	**Nonavalent HPV**	**Meningococcal**
**Characteristic**	**Category**					
	Total	496	489	473	325	290
Syphilis	Negative	496 (100.0%)	489 (100.0%)	471 (99.6%)	325 (100.0%)	289 (99.7%)
	Positive	0	0	1 (0.2%)	0	1 (0.3%)
	Not Done	0	0	1 (0.2%)	0	0
Chlamydia trachomatis	Negative	438 (88.3%)	434 (88.8%)	413 (87.3%)	293 (90.2%)	252 (86.9%)
	Positive	58 (11.7%)	55 (11.2%)	60 (12.7%)	32 (9.8%)	38 (13.1%)
Neisseria gonorrhoeae	Negative	488 (98.4%)	480 (98.2%)	466 (98.5%)	322 (99.1%)	285 (98.3%)
	Positive	8 (1.6%)	9 (1.8%)	7 (1.5%)	3 (0.9%)	5 (1.7%)
HSV-2	Negative	407 (82.1%)	387 (79.1%)	375 (79.3%)	264 (81.2%)	226 (77.9%)
	Positive	88 (17.7%)	102 (20.9%)	98 (20.7%)	60 (18.5%)	64 (22.1%)
	Indeterminate	1 (0.2%)	0	0	1 (0.3%)	0
Bacterial vaginosis*	Negative	415 (83.7%)	378 (77.3%)	378 (79.9%)	278 (85.5%)	239 (82.4%)
	Positive	81 (16.3%)	111 (22.7%)	95 (20.1%)	47 (14.5%)	51 (17.6%)
Trichomonas vaginalis	Negative	477 (96.2%)	468 (95.7%)	452 (95.6%)	315 (96.9%)	275 (94.8%)
	Positive	19 (3.8%)	21 (4.3%)	21 (4.4%)	10 (3.1%)	15 (5.2%)

*Nugent scores 7-10 were classified as BV positive and Nugent score 0-6 were classified as BV negative.
